# Regulatory Mechanism of SAC Content in Chloride Binding Characteristics of Ternary Repair Materials

**DOI:** 10.3390/ma19132862

**Published:** 2026-07-04

**Authors:** Xiang He, Mengdie Niu, Heng Zhou, Jingjing He, Honglin Xie, Cunbao Hu, Li Qian, Fangping Li

**Affiliations:** 1 PowerChina Northwest Engineering Co., Ltd., Xi’an 710100, China; helbert@xauat.edu.cn (X.H.);; 2 College of Materials Science and Engineering, Xi’an University of Architecture and Technology, Xi’an 710055, China; 3 Huaneng Lancang River Hydropower Inc., Kunming 650214, China; 4 College of Water Resources and Hydropower Engineering, Sichuan University, Chengdu 610065, China; 5 Dadu River Jinchuan Hydropower Construction Co., Ltd., Chengdu 610000, China

**Keywords:** OPC-GGBS-SAC, chloride binding capacity, sulfoaluminate cement (SAC), repair materials, chloride erosion

## Abstract

Corrosion of reinforcing steel and degradation of concrete caused by chloride penetration are the most critical forms of durability failure in marine environments. This requires that repair materials possess both high impermeability and stable chemical binding capacity. In this study, the impact patterns of sulfoaluminate cement (SAC) dosage on the chloride erosion durability of an OPC-GGBS-SAC ternary repair system were systematically evaluated. Through chloride ion binding capacity tests, electrical flux experiments, and microscopic analytical techniques including XRD, DTG and SEM-EDS, the synergistic regulation mechanisms of the dual functions of ‘physical barrier’ and ‘chemical binding’ in the composite material were elucidated. The findings show that the performance of the composite material was optimal at an SAC content of 10%. The electrical flux of composite materials at 28 d was 28.9% lower than that of the OPC system, whilst the chloride ion binding rate increased by 3.92%. Microstructural analysis indicates that an appropriate amount of SAC promoted the generation of ettringite (AFt) to optimize the early-age pore structure and stimulated the production of more C-S-H gel and AFm phases, thus synergistically enhancing impermeability and chemical binding capacity. When the SAC content exceeded 10%, excess gypsum inhibited the formation of AFm. Moreover, the concentration of early-stage hydration led to microdefects, resulting in a decline in durability. This study identifies the optimal dosage of SAC in the ternary system and clarifies the underlying mechanism, thereby providing a scientific basis for designing high-durability repair materials suitable for harsh ocean conditions.

## 1. Introduction

In marine environments, chloride penetration is the predominant factor causing the deterioration of reinforced concrete durability. This process destroys the passivation film on the steel surface, initiating and accelerating electrochemical corrosion. The resulting crystallization expansion pressure may also physically damage the concrete matrix, leading to cracking and spalling [1]. Consequently, cement-based materials used for repair and reinforcement must exhibit both high resistance to chloride penetration and strong chloride binding capacity. These dual capabilities are essential for ensuring the sustained stability of the repair interface and preventing the transport of corrosive agents.

Enhancing the resistance of cement-based materials to chloride ion primarily relies on the synergistic action of physical barrier and chemical solidification mechanisms. Among these, the physical barrier achieves effective blockage of chloride ion migration pathways by optimizing the pore structure of the matrix, refining pore size distribution, and reducing pore connectivity, thereby mitigating the corrosion rate of chloride ions [2,3]. Chemical solidification relies on the cementitious system to generate chloroaluminate phases such as Friedel’s salt through chemical reactions, stably binding chloride ions within the hydration products and thereby reducing the free chloride ion content in the system [4,5,6,7]. A ternary system composed of ordinary Portland cement (OPC), ground granulated blast-furnace slag (GGBS) and sulfoaluminate cement (SAC) integrates the late-stage stability of OPC, the progressive densification effect of GGBS and the early strength and microexpansive characteristics of SAC. This system has become a promising direction for high-performance repair materials [8,9]. Previous studies have shown that the early hydration of SAC produces large amounts of ettringite (AFt), together with the pozzolanic reactivity of GGBS, which generates a C-(A)-S-H gel with a low Ca/Si ratio. The synergistic effect of these two components can significantly reduce the porosity of the composite system, thus improving its resistance to ionic permeation [10]. Nevertheless, current research mainly focuses on the macroscopic impermeability and mechanical properties of the system, and there is still a lack of systematic interpretation of the chemical curing behavior of chloride ions in this system.

In terms of chemical solidification, existing research has shown that the chemical binding of chloride ions mainly depends on the active aluminum phase in the cementitious material. The increase in Al_2_O_3_ content in cement-based materials can effectively promote the formation of Friedel’s salts and enhance the chloride ion curing effect [11]. Due to its high Al_2_O_3_ content, GGBS has been shown to significantly increase the number of chemical-bound chlorides in the system and to promote the formation of Friedel’s and Kuzel’s salts [9,12]. Furthermore, GGBS can also facilitate the production of C-(A)-S-H gel by incorporating Al into the C-S-H structure, thereby significantly improving both the quality and quantity of hydration products and enhancing the physical adsorption capacity of gel-like hydration products for chloride ions [13,14]. Yu [15] found that the optimal chloride ion binding effect can be achieved by adding 20 wt.% GBBS, whereas Zhang [16] found that the bound chloride ion content in cement-based materials first increases and then decreases as the GGBS content increases, with the optimal replacement rate falling within the range of 40–60%. In addition, excessively high GGBS content can degrade the mechanical properties of the system [17]. The differences observed in the above studies are primarily due to variations in the activity of GGBS from different sources and with different compositions. The previous study by this research group [18] confirmed that GGBS can chemically bind chloride ions, and its pozzolanic effect can improve the content of C-S-H gel and refine the pore structure. When the content is 30%, the comprehensive performance is the best.

Regarding the role of SAC in chloride ion binding, research by Cheng et al. [19] on anhydrous calcium sulfoaluminate (C_4_A_3_Š) indicates that gypsum content is a key parameter for controlling the ratio of AFm to AFt, and it directly affects the chloride binding performance of SAC under conditions of external chloride penetration and internal chloride incorporation. Meanwhile, the AFm phase present in SAC hydration products also provides important binding sites for chlorides [20]. Furthermore, layered double hydroxides (LDHs), as synthetic chloride adsorbents, have been confirmed to effectively enhance the chloride binding ability of cement-based materials, thus inhibiting steel corrosion [21]. OPC-SAC-GGBS composite materials with added LDHs can achieve a maximum adsorption capacity of 43.5 mg/g. The chloride ion binding capacity of the system is evidently unaffected by prolonged erosion [22].

In terms of the OPC-SAC binary system, research by Xin et al. [23] indicates that the C_4_A_3_Š mineral in SAC and the C_3_S mineral in OPC mutually enhance each other during co-hydration, thereby accelerating the hydration and setting of the blended cement [20,24]. A study by Zhou [25] showed that the comprehensive performance of OPC-SAC composite cementitious materials is optimal when the SAC content is 15%. A study by Wang [26] indicated that with an increase in SAC dosage, the mechanical properties of the OPC-SAC composite system first increase and then decrease, and the optimal dosage is 10%~20%. Moreover, the addition of SAC can effectively alleviate the negative impact of high GGBS content on the mechanical properties of the OPC-GGBS system, providing a research basis for the synergistic optimization of the properties of ternary systems [8]. Adding an appropriate amount of SAC can effectively improve the compressive strength and bond strength of the OPC-SAC-GGBS system and suppress its shrinkage deformation [27]. In summary, existing research has largely clarified the mechanical property evolution law and the chloride ion binding mechanism of the OPC-SAC system and the OPC-GGBS system. However, for the OPC-GGBS-SAC ternary system, there remains limited quantitative research and in-depth mechanistic analysis regarding how the SAC content dynamically influences the hydration process, product composition and microstructure, and consequently synergistically regulates physical barrier and chemical curing performance.

Therefore, this study focuses on SAC content as a key parameter and systematically investigates its role in the chloride binding behavior of composite repair materials. The underlying binding mechanisms at varying SAC contents are elucidated by combining chloride binding capacity measurements with microstructural analyses (XRD, DTG, SEM EDS). The findings are expected to provide theoretical support for the development of durable repair materials designed for severe marine conditions.

## 2. Materials and Methods

### 2.1. Materials

The binder employed in this experiment includes: 52.5R ordinary Portland cement (OPC), 42.5 sulfoaluminate cement (SAC), and S95 ground granulated blast-furnace slag (GGBS). OPC, SAC and GGBS meet the Chinese standards GB/T 18046-2017, GB 175-2023 and GB/T 20472-2026, respectively [28,29,30]. Their chemical constituents and physical properties are summarized in Table 1, Table 2 and Table 3, respectively. To improve workability, a polycarboxylate superplasticizer (PCE) with a water-reducing rate of 25% was added. The fluidity of mortar was tested according to the national standard GB/T 50448-2015 [31]. For the fine aggregate, a mixture of two gradations of quartz sand was selected (0.2–0.5 mm and 0.5–1.0 mm, in a mass ratio of 4:6).

### 2.2. Sample Preparation

Based on the results of preliminary tests, the OPC-GGBS composite system exhibits optimal mechanical properties and chloride ion binding capacity when the GGBS content is 30% [27]. Considering that either GGBS or SAC consumes Ca(OH)_2_, a severe reduction in OPC content would hinder the maintenance of alkalinity in the OPC-GGBS-SAC system, thereby potentially impairing the steel reinforcement protection properties of the repair mortar. Therefore, SAC was introduced to modify a mixture of OPC and GGBS in a 70:30 ratio, with SAC replacing OPC and GGBS at a 7:3 mass ratio. Paste specimens were used to measure chloride ion content, with a PCE dosage of 0.2 wt.% of the binder. Mortar specimens were used to determine the electric flux of the repair material. The proportioning design of the OPC-GGBS-SAC mortar and paste samples is shown in Table 4 and Table 5, respectively.

### 2.3. Test Methods

#### 2.3.1. Determination of Chloride Ion Content in Cementitious Materials

**Figure 1:**
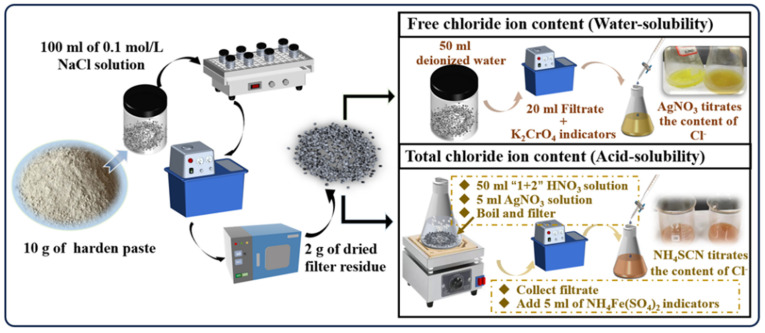
Test flow of chloride ion adsorption and binding capacity.

The procedure for evaluating the chloride adsorption and binding capacity of the OPC-GGBS-SAC cementitious material is shown in Figure 1. After 3, 7, and 28 d of curing, powder samples were drilled from the paste specimens, ground and sifted with a 75 μm sieve, and then oven-dried at 40 °C in a vacuum desiccator. Subsequently, 10 g of the dried powder was immersed in 100 mL NaCl solution of 0.1 mol/L for a specified period, filtered and dried [32]. The free chloride (*C_f_*) and total chloride (*C_t_*) amounts of the immersed powder were determined according to the standard method GB/T 176-2025 [33]. The bound chloride (*C_b_*) content was determined through the subtraction of *C_t_* and *C_f_*, and the chloride binding ratio was defined as *C_b_*/*C_t_* [34]. The *C_f_* and *C_t_* contents were calculated using Equations (1) and (2), respectively:

(1) $$C_{f}=\frac{C_{{\mathrm{AgNO}}_{3}}\times V_{3}\times 0.03545}{G\times \frac{V_{2}}{V_{1}}}\times 100$$

where *C_f_* is the mass percentage of water-soluble (free) chloride ion (%); $C_{{\mathrm{AgNO}}_{3}}$ is the AgNO_3_ titrant concentration (mol/L); $V_{3}$ is the consumed volume of nitrate silver solution during titration (mL); *G* is the sample mass (g); $V_{2}$ is the titration filtrate volume (mL); and $V_{1}$ is the volume of deionized water used for chloride extraction (mL).

(2) $$C_{t}=\frac{T_{{\mathrm{Cl}}^{-}}\times V_{{\mathrm{AgNO}}_{3}}\times \left(V_{1}-V_{2}\right)}{V_{1}\times m\times 1000}\times 100$$

where *C_t_* is the mass percentage of acid-soluble (total) chloride ion (%); $T_{{\mathrm{Cl}}^{-}}$ is the titer of silver nitrate with respect to chloride ion (mg/L), calculated as *T*_Cl_^−^ = *C*_AgNO3_ × 35.45; $V_{{\mathrm{AgNO}}_{3}}$ is the volume of AgNO_3_ solution used for back-titration (mL); $V_{1}$ is the volume of NH_4_SCN solution consumed by the blank (mL); $V_{2}$ is the amount of NH_4_SCN solution utilized during titration (mL); and *m* is the sample mass (g).

#### 2.3.2. Rapid Chloride Permeability Test

The electric flux was conducted in compliance with the standard GB/T 50082-2024 [35]. After curing for 28 d, the specimens were dried at 40 °C for 48 h, and then subjected to vacuum saturation. The specimens were subsequently placed into specimen chambers, with a 3.0 wt.% NaCl solution and a 0.3 mol/L NaOH solution poured into the negative and positive terminals, respectively. Once the setup was complete, continuous DC voltages of 60 ± 0.1 V were supplied. The electric flux (C) passing through each sample over a 6 h period was automatically recorded by the testing apparatus. The average value obtained from three specimens was recorded to represent the final electric flux for each group.

#### 2.3.3. Microstructure

At specified curing ages, block or powder samples were taken from designated locations of the paste specimens. The sample was immediately placed in anhydrous ethanol to stop hydration, then ground and filtered by a 75 µm sieve. The resulting powder was dried for 48 h at 40 °C under vacuum, and the crystal compositions were analyzed by X-ray diffraction (XRD). Thermogravimetric analysis (DTG) was carried out over a thermal range of 25~900 °C at a rising temperature speed of 10 °C/min under a protective nitrogen atmosphere. In addition, paste specimens were immersed in NaCl solution and deionized water until predetermined ages. After removal of the surface layer (approximately 1 mm), block samples were obtained and then dried. The microstructure was examined by scanning electron microscopy (SEM-EDS).

## 3. Results and Discussion

### 3.1. Effect of Hydration Age on Chloride Binding Capacity of the OPC-GGBS-SAC

**Figure 2:**
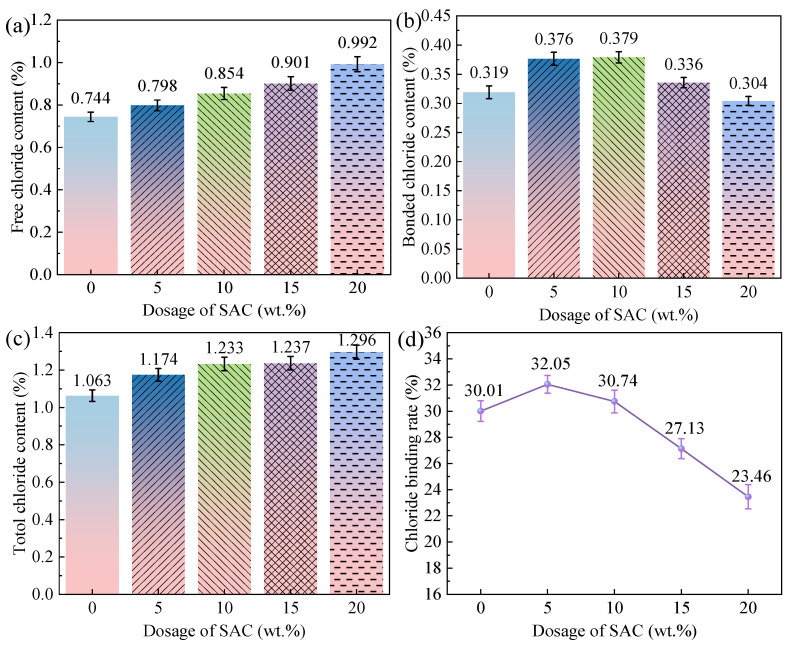
Cl^−^ binding capacity of OPC-GGBS-SAC after 3 d immersion and hydration for 3 d. (**a**) *C_f_*; (**b**) *C_b_*; (**c**) *C_t_*; (**d**) Cl^−^ binding rate.

**Figure 3:**
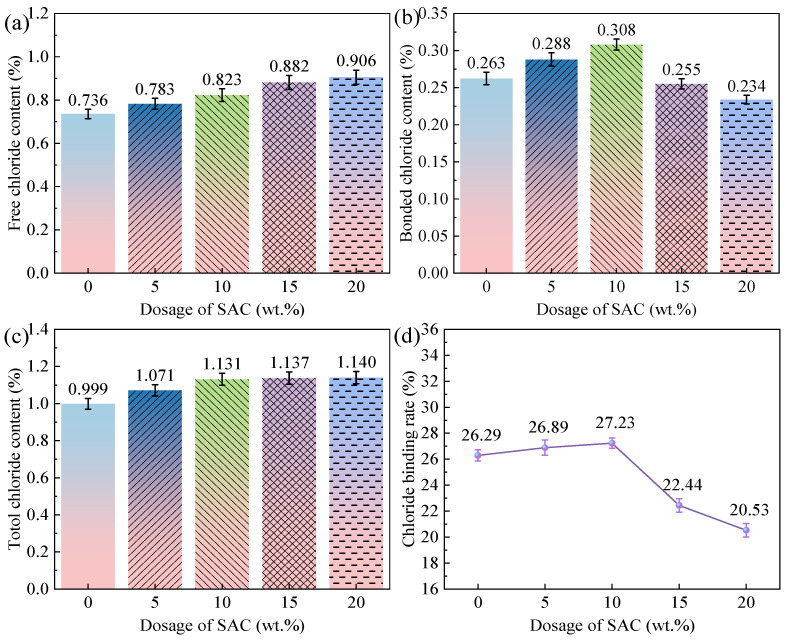
Cl^−^ binding capacity of OPC-GGBS-SAC after 3 d immersion and hydration for 7 d. (**a**) *C_f_*; (**b**) *C_b_*; (**c**) *C_t_*; (**d**) Cl^−^ binding rate.

**Figure 4:**
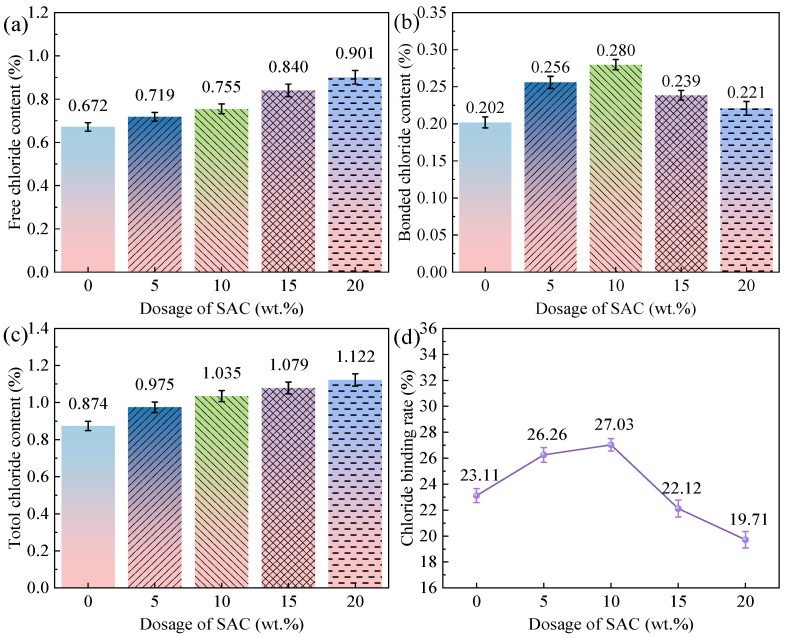
Cl^−^ binding capacity of OPC-GGBS-SAC after 3 d immersion and hydration for 28 d. (**a**) *C_f_*; (**b**) *C_b_*; (**c**) *C_t_*; (**d**) Cl^−^ binding rate.

The chloride binding capacity of the OPC-GGBS-SAC system at different hydration ages is shown in Figure 2, Figure 3 and Figure 4. As hydration age increased, the *C_f_*, *C_b_*, and chloride binding ratio of the s matrix progressively declined. This decline is attributed to the encapsulation of chloride ions by newly formed hydration products, and cement paste at early ages can absorb or bind more chlorides. During the early hydration stage, the system contains a significant amount of the AFm phase, whose layered structure efficiently immobilizes Cl^−^ as Friedel’s salt through anion exchange [36]. Meanwhile, the initial porosity was relatively high, and hydration compounds like C–S–H gels provided a large specific surface area for physical adsorption. As hydration progressed, the pore structure gradually became denser, reducing the number of physical adsorption sites. More importantly, the concentration of OH^−^ in the pore fluid continued to rise. Under highly alkaline conditions, pre-formed Friedel’s salt may undergo dechlorination, leading to a decline in stability and the subsequent release of some bound Cl^−^ [37]. Therefore, as the age of hydration increased, the ability to bind chloride ions decreased.

As the SAC content increased, the *C_b_* and chloride binding ratio of the composite system first increased and then decreased, whereas the *C_f_* and *C_t_* continuously rose. For specimens with a hydration period of 3 d (Figure 2), the highest *C_b_* and chloride binding ratio were achieved at an SAC content of 5%. For specimens at 7 and 28 d (Figure 3 and Figure 4), the optimal values were observed at an SAC content of 10%. The differences in chloride binding capacity of the composite system at varying SAC contents arise from the amount of hydration products formed. In the early hydration stage, a small quantity of SAC promoted the hydration reaction, increased the degree of hydration, and generated more hydration products, which include the AFm that binds chloride ions. However, when the SAC content further accumulated, the degree of hydration of the system was adversely affected, resulting in a decline in chloride binding efficiency.

As hydration proceeds, OPC and GGBS in the OPC-GGBS-SAC ternary materials underwent extensive hydration. In particular, the Al phases in GGBS contributed to an increased formation of AFm, and the optimal content of SAC shifted from 5% to 10%. This indicates that, at later stages, the active Al_2_O_3_ continuously released from GGBS reacted with Ca(OH)_2_ in the system, providing additional potential for AFm formation. At an appropriate SAC content of 10%, the aluminum and calcium sources supplied by SAC synergistically interacted with the contribution from GGBS, causing the total AFm generation to reach a peak [38]. Compared with the control group (S0), the chloride binding ratio at a hydration age of 28 d increased by 3.92% when the SAC content was 10%.

AFt was the main hydration compound of SAC. Although there was a mechanism for the conversion of AFt to AFm, significant amounts of AFm were only formed when there was a shortage of CaSO_4_ in the setting system. To prevent SAC from undergoing strength loss in the later stages, most SAC products currently available were supplemented with sufficient gypsum to inhibit the transformation of AFt phases. Specifically, low-content SAC (5%) provided an abundance of aluminate and calcium ions in the early stages, promoting AFm formation. Meanwhile, the sulfate ions introduced by SAC were not yet excessive, so the transformation of AFt into AFm was not completely suppressed [39,40]. When the SAC content was excessively high, the substantial gypsum incorporated into commercial SAC products to prevent late-stage strength regression dissolved and released a large amount of SO_4_^2−^. These sulfate ions strongly competed for the anion sites between AFm interlayers and hindered the transformation of AFt to AFm, thereby diminishing the ability to bind chemical chlorides [41]. Consequently, the decline in chloride binding capacity at excessively high SAC contents was primarily attributed to the replacement of OPC and GGBS by SAC, thereby reducing the amount of AFm formed. Therefore, when SAC is used to modify composite systems, it is particularly important to keep its content within a reasonable range to enhance the chloride fixation ability of the ternary system.

### 3.2. Effect of Exposure Time on Chloride Binding Capacity of the OPC-GGBS-SAC

**Figure 5:**
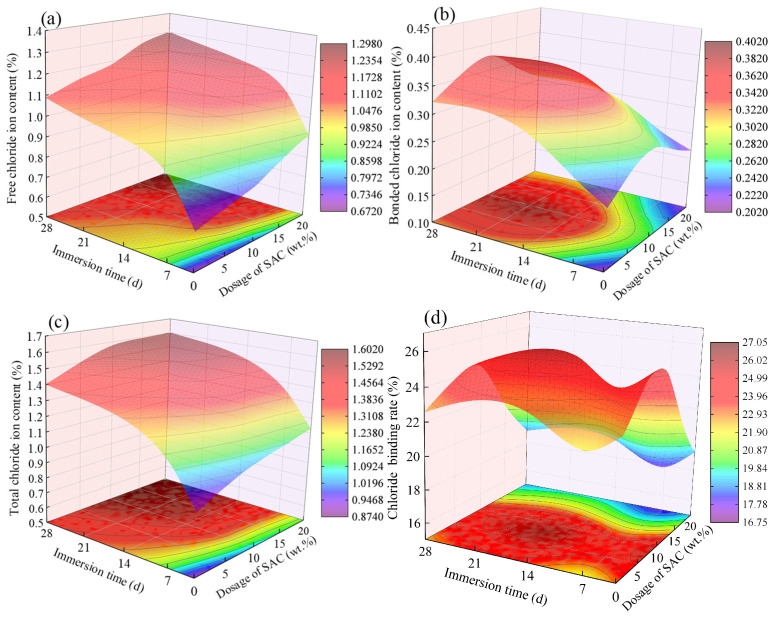
Cl^−^ binding capacity of OPC-GGBS-SAC at different immersion times. (**a**) *C_f_*; (**b**) *C_b_*; (**c**) *C_t_*; (**d**) Cl^−^ binding rate.

The chloride binding capacity of the OPC-GGBS-SAC ternary materials at different immersion times is presented in Figure 5, and the relevant data are listed in Table 6. As shown in Figure 5a,c, the *C_f_* and *C_t_* adsorbed by the composite system gradually increased with prolonged immersion time, reaching a plateau after 21 d of immersion. In addition, as the SAC content increased, both C*_f_* and C*_t_* also rose gradually. When the SAC content ranged from 0 to 10%, the increase in C*_f_* was relatively pronounced. Once the SAC content exceeded 10%, the *C_f_* values remained largely unchanged. During long-term immersion, SAC in the composite system hydrated preferentially, and the ongoing hydration of OPC and GGBS gradually increased the mass of hydration compounds. When the SAC proportion was within the appropriate range, it contributed to facilitating the formation of hydration products, thereby increasing the number of active adsorption sites for chloride ions. However, this beneficial effect was weakened when the SAC content exceeded the optimal level.

This conclusion is also reflected in Figure 5b,d. With increasing SAC content, the *C_b_* values in the composite system at different immersion times consistently showed a tendency to rise initially and then decrease, reaching the highest at an SAC content of 10%. As the same quantity of cementitious materials was used, the overall quantity of hydration products was comparable across all sample groups following prolonged hydration; a higher SAC content reduced the proportions of OPC and GGBS in the system. This reduction may decrease the content of AFm, which serves a critical function in chemically binding chlorides within the hydration products.

As the soaking time increased, the chloride binding ratio first increased and then decreased. This trend can be attributed to the fact that, as immersion time is prolonged, the times at which free and bound chloride ions reach adsorption saturation differ, leading to an inconsistent increase in *C_b_* and *C_t_*. After 21 d of immersion, the chloride binding ratio declined. This was because the chemical binding capacity of the composite system approached saturation, while *C_t_* continued to increase slowly. Across different immersion times, the highest chloride binding ratio was achieved at SAC contents ranging from 5% to 10%, indicating that the OPC-GGBS-SAC ternary materials exhibited optimal chloride binding capacity within this dosage range of SAC.

### 3.3. Effect of SAC Content on Chloride Penetration Resistance of OPC-GGBS-SAC Repair Mortar

**Figure 6:**
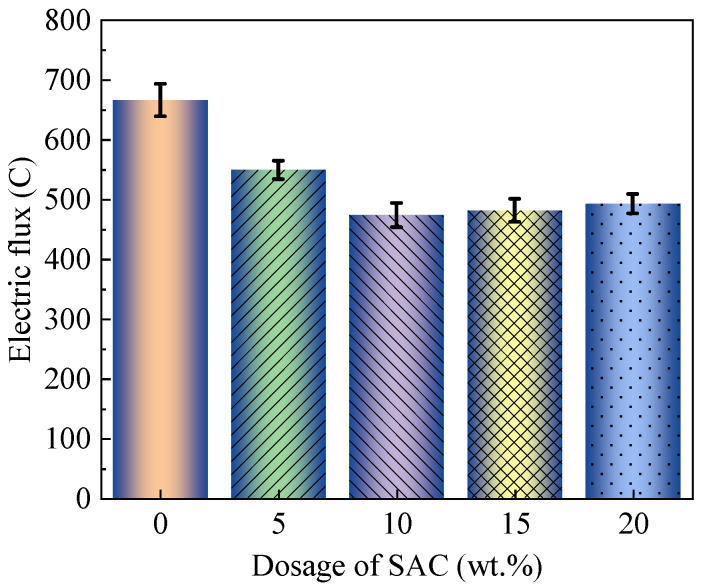
Influence of SAC on electric flux of OPC-GGBS-SAC mortars.

The effect of SAC content on the electric flux of ternary-system mortar is shown in Figure 6. As the SAC proportion increased, the electric flux of the mortar first decreased and then increased. At an SAC proportion of 10%, the electrical flux reached its lowest value, indicating the optimal chloride penetration resistance, and the electrical flux was reduced by 28.9% compared with the control group. This improvement is because of the substantial optimization of the pore structure. At this optimal dosage, AFt formed during SAC hydration effectively filled the early-stage capillary pores, creating a dense skeleton. Meanwhile, the optimal proportion of SAC accelerated the hydration of OPC and activated GGBS, promoting the generation of a large amount of C-S-H gel with a low Ca/Si ratio. This gel further filled the nanopores, achieving multiscale pore refinement and significantly increasing the tortuosity of ion migration [42,43]. Under these conditions, the SO_4_^2−^ concentration in the system was moderate, which not only stabilized AFt but also did not hinder the long-term pozzolanic reaction of GGBS.

When the SAC content was relatively low (5%), the amount of AFt formed was insufficient to adequately fill the early-stage pores, resulting in a relatively loose structural skeleton and limiting the potential for maximizing densification in the later stages. When the SAC content exceeds 10%, the excessively rapid early hydration of the system may lead to a reduction in overall hydration and an increase in porosity, elevating the risk of microcracking. Moreover, this may lead to localized aggregates of AFt and the formation of weak interfaces, compromising structural uniformity. Therefore, the chloride penetration resistance of the mortar specimens declined.

### 3.4. Chloride Binding Mechanism of OPC-GGBS-SAC Composite System

#### 3.4.1. XRD Analysis

**Figure 7:**
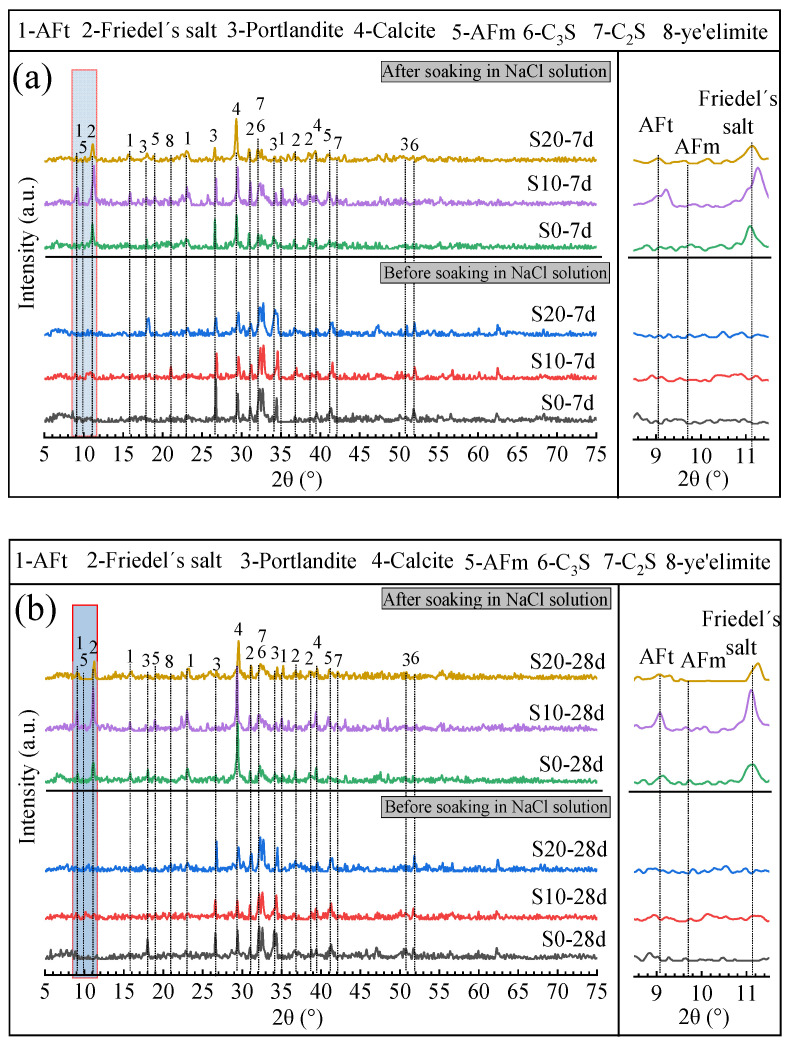
XRD patterns of OPC-GGBS-SAC before and after soaking in NaCl solution: (**a**) 7 d; (**b**) 28 d.

Figure 7 displays the XRD patterns of the OPC-GGBS-SAC ternary system before and after soaking in NaCl aqueous solution. For the early-stage samples (Figure 7a), prior to immersion, the addition of SAC increased the peak intensity of AFt diffraction, while reducing that of the Portlandite diffraction peak. The main hydration product of SAC was AFt. As the proportion of SAC increased, the proportion of OPC decreased, resulting in reduced Ca(OH)_2_ formation. In addition, the occurrence of ye’elimite was observed in the early-stage samples, indicating that part of the SAC remained unhydrated after 7 d of curing.

Following 7 d of soaking in NaCl solution, the diffraction peak intensity of Friedel’s salt increased significantly, with S10 showing a higher intensity than S20. This pattern is compatible with the measured bound chloride concentrations in the two groups of samples, suggesting that S10 generated more AFm during hydration, thus enabling it to bind more chlorides. On one hand, when the SAC content exceeded the appropriate range, the formation of AFm was reduced due to the decreased amount of GGBS in the system. On the other hand, at the optimal SAC content, the overall degree of hydration of the system was higher, and the quantity of hydration products also increased. After immersion, the diffraction peak intensity of Portlandite increased, while that of C_3_S and C_2_S decreased, indicating that the specimen underwent further hydration during the immersion period.

For the samples hydrated for 28 d (Figure 7b), the peak of ye’elimite nearly diminished, indicating that SAC in the system had been almost completely hydrated. After immersion, distinct diffraction peaks of Friedel’s salt were noted, with the peak intensity of S10 being higher than those of S0 and S20. This trend is consistent with the pattern of how SAC content affects the concentration of combined chlorides. Meanwhile, the diffraction peak corresponding to Portlandite is significantly weakened. This is due to the pozzolanic effect of GGBS during the later hydration stage, which consumed Ca(OH)_2_. The consequent generation of additional C-S-H gel further densified the composite system.

#### 3.4.2. TG Analysis

**Figure 8:**
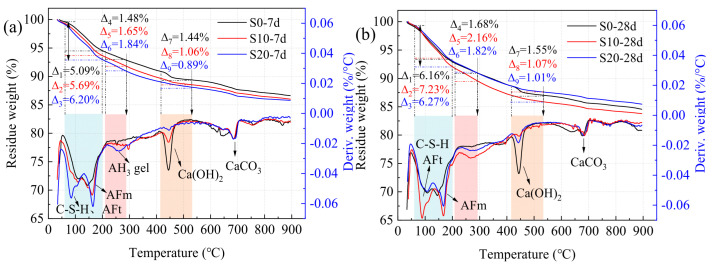
DTG curves of OPC-GGBS-SAC at different hydration stages: (**a**) 7 d; (**b**) 28 d.

The DTG curves of the OPC-GGBS-SAC ternary system are illustrated in Figure 8. For the samples hydrated for 7 d (Figure 8a), the addition of SAC favored the development of hydration products including C-S-H, AFt, AFm, and AH_3_ gel. Compared with the control group, the thermal weight losses of S10 and S20 in the 60~200 °C range improved by 0.60% and 1.11%, respectively. This is because the primary hydration compound of SAC was AFt, and the hydration reaction occurred mainly at early stages. Furthermore, SAC enhanced the hydration reaction of OPC and GGBS, thereby generating more C-S-H, AH_3_ gel and other phases. In the 420~530 °C range [44], the thermal weight losses of S10 and S20 decreased by 0.38% and 0.55%, respectively. This reduction is due to the decreased proportion of OPC in the ternary materials with increasing SAC proportion. Since Ca(OH)_2_ was generated solely by the hydration of OPC, the quantity of Ca(OH)_2_ produced consequently decreased.

In the samples hydrated for 28 d (Figure 8b), the thermal weight losses of S10 and S20 in the 60~200 °C range increased by 1.07% and 0.11%, respectively. This increase may be ascribed to two reasons. Firstly, the addition of SAC increased the amount of AFt formed in the system. Secondly, a minor percentage of SAC enhanced the degree of hydration of the composite system, thereby increasing the total amount of hydration products. Similarly, in the 210~290 °C range, the thermal weight losses of S10 and S20 increased by 0.48% and 0.14%, respectively. When the SAC proportion was above the optimal level, the reduction in OPC proportion and the encapsulation of unhydrated cement particles resulting from quick hydration of SAC led to a decrease in the formation of hydration products such as C-S-H and AH_3_ gel. In the 420~530 °C range, the thermal weight losses of S10 and S20 decreased by 0.48% and 0.54%, respectively, which is primarily due to the reduced proportion of OPC in the cementitious system.

**Figure 9:**
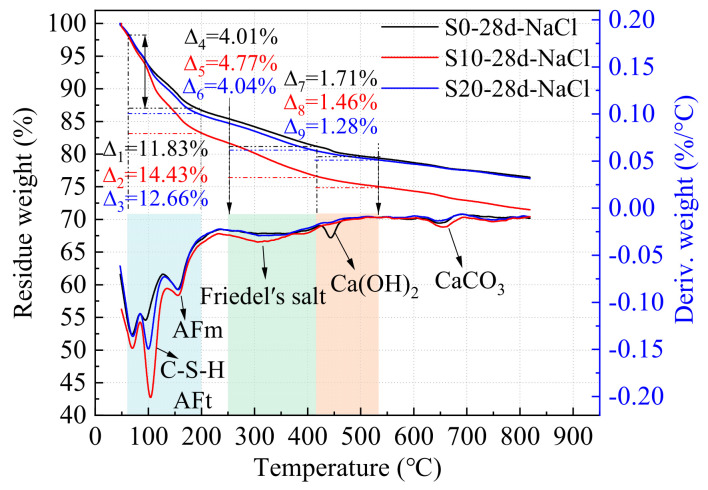
DTG curves of OPC-GGBS-SAC after immersion in NaCl.

Figure 9 presents the DTG curves of the ternary system after 7 d of immersion in NaCl solution. In the temperature range of 250~420 °C, the thermal weight losses of S10 and S20 increased by 0.76% and 0.03%, respectively. The increase in Friedel’s salt was more noticeable in S10, whereas the increase in Friedel’s salt in S20 was not significant [45]. This indicates that at an SAC content of 10%, the composite system immobilized a greater number of chlorides, which is consistently supported by the results on chloride binding capacity at different SAC contents shown in Figure 2, Figure 3, Figure 4 and Figure 5. A moderate amount of SAC favored the formation of the AFm phase. When the SAC proportion exceeded 10%, the reduction in OPC proportion and the decrease in the degree of hydration caused diminished the formation of AFm, thereby weakening the chloride binding capacity of the system.

#### 3.4.3. SEM-EDS Analysis

**Figure 10:**
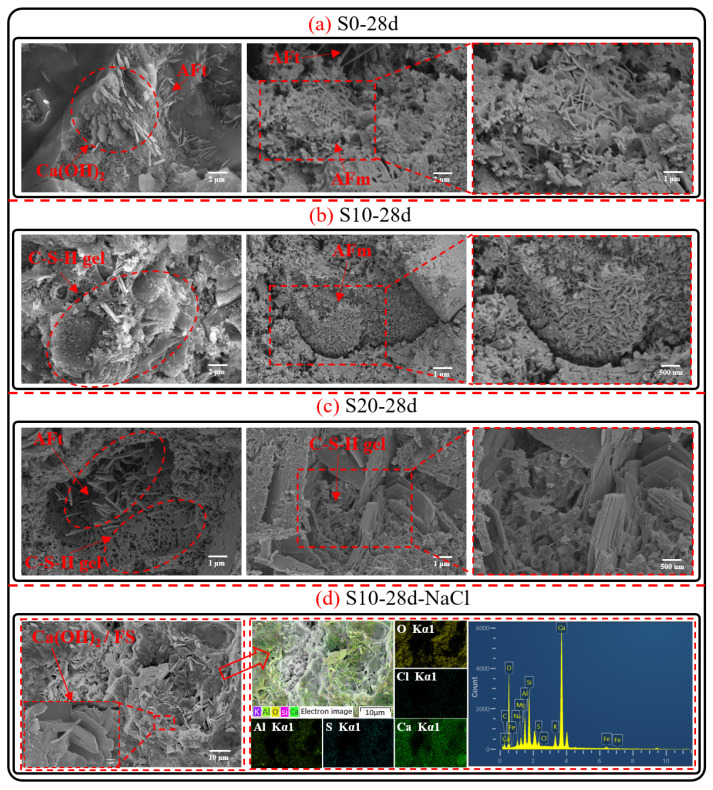
SEM-EDS images of OPC-GGBS-SAC.

Figure 10 depicts the SEM-EDS images of the ternary system. In S0 (Figure 10a), hexagonal plate-like portlandite crystals were stacked together, exhibiting complete crystal morphology and distinct growth orientation. Needle-like AFt crystals and petal-like AFm crystals were encapsulated by C-S-H. In S10 (Figure 10b), C-S-H gel permeated the pores, resulting in a generally dense structure. Extensive petal-shaped AFm crystals were also observed on the surface of underhydrated cement particles. These AFm crystals grew around the cement particles, generating expansion stress that induced microcracks in the surrounding matrix.

In S20 (Figure 10c), AFt grew in an interpenetrating manner within the C-S-H gel. Notably, C-S-H gel in S20 appeared loose and exhibited a honeycomb-like morphology. The presence of loose C-S-H gel was also observed in the pores formed by stacked Ca(OH)_2_ crystals. In contrast, in S0 and S10, the pores formed a relatively dense structure after being filled with C-S-H gel, whereas in S20, there were still a significant number of voids within the regions where the C-S-H gel had accumulated. SAC hydrates faster than OPC. Even by the end of the intense exothermic phase, OPC in the ternary system had not yet begun to hydrate. This indicates that a large amount of AFt had already formed during the early hydration age (before the onset of OPC hydration), and that the AFt formed numerous voids through disordered interlocking in the pore space. Once OPC began to hydrate and produce C-S-H gel, the chaotically interwoven AFt occupied most of the pore channels, hindering the migration of C-S-H gel toward pores and defects. Hence, in the voids formed by the interlocking or stacking of AFt and Ca(OH)_2_ crystals, C-S-H gel was unable to fill these pores to create a compact structure, giving rise to the observed honeycomb-like C-S-H and loose microstructure. Furthermore, some unhydrated cement particles were encapsulated by AFt, reducing the degree of hydration of the ternary system and thereby decreasing the amount of C-S-H gel formed. This further weakened the pore-filling effect of C-S-H gel.

After immersion in NaCl solution for 7 d (Figure 10d), numerous irregular plate-like crystals were observed in the S10 sample. Their morphology differed significantly from that of Ca(OH)_2_ in the sample before immersion. This may be due to calcium leaching of Ca(OH)_2_ during immersion, which altered the crystal morphology into irregular forms. Alternatively, it may be the result of Friedel’s salt formed when AFm in the ternary system combined with Cl^−^ [46]. EDS analysis of this region revealed that the elements present in the scanned area were primarily Ca, O, Si, Al, S and Cl. Among these, Al, Ca, and Cl were highly enriched at the locations corresponding to the irregular plate-like crystals, indicating the presence of Friedel salts in this region.

## 4. Conclusions

(1) SAC effectively reduced the chloride penetration resistance of the OPC-GGBS-SAC ternary system. At an SAC proportion of 10%, the electric flux reached its minimum value, which decreased by 28.9% compared to the blank group.
(2) A low percentage of SAC accelerated the hydration process of the ternary system, enhanced the degree of hydration, and generated more hydration products, including the AFm phase, which binds chlorides. When the SAC content was 10%, the chloride binding ratio of the composite system reached its maximum, representing an increase of 3.92% compared with the control group. When the SAC content further increased beyond this level, the extent of hydration of the ternary materials was adversely affected, causing a decline in chloride binding capacity.
(3) When the SAC content exceeded 10%, the primary reason for the reduction in chloride ion binding ability was that the SAC replaced the OPC and GGBS in the system, which reduced the amount of AFm formed. Therefore, when using SAC to modify composite systems, it is particularly important to keep the dosage within a reasonable range to enhance the capability to bind chloride ions of the ternary materials.

Adopting the perspective of synergistic interplay between “physical blocking” and “chemical binding”, the regulatory mechanism and critical threshold of SAC regulating the chloride durability of the OPC-GGBS-SAC ternary system were systematically elucidated in this study. The SAC content determined the pore structure and chloride binding capacity of the material through three key mechanisms: controlling the early-stage nucleation and growth of AFt, influencing the long-term transformation and formation of AFm, and regulating the densification process of C-S-H gel. The study identified 10% as the critical threshold for SAC content. At this level, the chloride ion binding rate of the OPC-SAC-GGBS system after 28 d of hydration under standard curing conditions was 27.03%, and the electrical flux was 473.53 C. Below this level, the optimization of the pore structure and the potential for chemical binding are not fully activated. Above this threshold, a series of interconnected issues, including the inhibition of AFm formation by excessive gypsum, an imbalance in the proportions of cementitious phase, and microdefects induced by concentrated early hydration, leads to a simultaneous decline in chloride binding ability and resistance to chloride permeation. The above conclusions provide a crucial theoretical basis and practical guidance for the precise design and performance optimization of durable repair materials intended for harsh marine environments.

Although the powder adsorption method can more accurately evaluate the effect of SAC dosage on the chloride ion binding ability of the OPC-GGBS-SAC system, this method itself ignores the influence of the actual pore structure of bulk materials. Therefore, in order to better understand the chloride ion adsorption and binding behavior of OPC-GGBS-SAC composite repair materials in real service environments, future research should adopt long-term immersion tests to determine the chloride ion content at different penetration depths and analyze its diffusion behavior, thereby providing a more comprehensive understanding of the chloride ion transport mechanisms and durability performance of the OPC-GGBS-SAC system in real-world service environments.

## Figures and Tables

**Table 1:** The chemical composition of cementitious materials (wt.%).

	CaO	Al_2_O_3_	SiO_2_	Fe_2_O_3_	MgO	SO_3_	K_2_O	Na_2_O	LOI
OPC	63.23	5.29	20.38	2.98	2.11	2.46	0.37	0.15	3.03
SAC	42.25	36.46	6.86	2.20	1.33	8.82	0.18	0.22	1.25
GGBS	40.81	18.46	31.55	2.42	4.13	2.05	0.24	0.13	0.21

**Table 2:** The physical properties of cement.

	Specific Surface Area (m^2^/kg)	Setting Time (min)		Compressive Strength (MPa)			
		Initial	Final	1 d	3 d	7 d	28 d
OPC	362	168	213	/	36.2	40.3	55.7
SAC	350	30	65	30.5	/	45.0	47.9

**Table 3:** The physical properties of GGBS.

Density (kg/m^3^)	Specific Surface Area (m^2^/kg)	Activity Index (%)		Flowability Ratio (%)
		7 d	28 d	
2875	462	79	98	102

**Table 4:** Mix proportion of OPC-GGBS-SAC mortars.

Mix ID	OPC (g)	GGBS (g)	SAC (g)	S (g)	PCE (g)	W (g)	Initial Fluidity (mm)
S0	700	300	0	1000	4.00	230	330
S5	665	285	50	1000	4.00	230	330
S10	630	270	100	1000	4.00	230	325
S15	595	255	150	1000	4.00	230	325
S20	560	240	200	1000	4.00	230	330

**Table 5:** Mix proportion of OPC-GGBS-SAC paste specimens.

Mix ID	OPC (g)	GGBS (g)	SAC (g)	PCE (g)	W (g)
S0	700	300	0	2.00	230
S5	665	285	50	2.00	230
S10	630	270	100	2.00	230
S15	595	255	150	2.00	230
S20	560	240	200	2.00	230

**Table 6:** Numerical data table of Cl^−^ binding capacity of OPC-GGBS-SAC at different immersion times.

Immersion Time (d)	Dosage of SAC (%)	C*_f_* (%)	C*_b_* (%)	C*_t_* (%)	Cl^−^ Binding Rate (%)
3	0	0.6721	0.2021	0.8741	23.1121
3	5	0.7191	0.2561	0.9751	26.2564
3	10	0.7553	0.2797	1.0351	27.0285
3	15	0.8404	0.2386	1.0791	22.1158
3	20	0.9001	0.2211	1.1211	19.7145
7	0	0.9026	0.2316	1.1342	20.4188
7	5	0.9341	0.2799	1.2141	23.0597
7	10	1.0079	0.2914	1.2993	22.4259
7	15	1.0571	0.2501	1.3071	19.1278
7	20	1.1228	0.2262	1.3491	16.7692
14	0	0.9941	0.3101	1.3041	23.773
14	5	1.0071	0.3511	1.3581	25.8468
14	10	1.0861	0.4011	1.4871	26.9671
14	15	1.1751	0.3211	1.4961	21.4572
14	20	1.2075	0.2975	1.5051	19.7664
28	0	1.0861	0.3131	1.3991	22.3731
28	5	1.1491	0.3531	1.5021	23.5021
28	10	1.1791	0.4021	1.5811	25.4269
28	15	1.2691	0.3231	1.5921	20.2889
28	20	1.2981	0.3031	1.6011	18.9257

## Data Availability

The original contributions presented in this study are included in the article. Further inquiries can be directed to the corresponding authors.
